# Genotypic Characterization of Antimicrobial Resistant *Salmonella* spp. Strains from Three Poultry Processing Plants in Colombia

**DOI:** 10.3390/foods10030491

**Published:** 2021-02-25

**Authors:** Alejandra Ramirez-Hernandez, Ana K. Carrascal-Camacho, Andrea Varón-García, Mindy M. Brashears, Marcos X. Sanchez-Plata

**Affiliations:** 1 Animal and Food Sciences Department, Texas Tech University, Lubbock, TX 79409, USA; mindy.brashears@ttu.edu (M.M.B.); marcos.x.sanchez@ttu.edu (M.X.S.-P.); 2 Microbiology Department, Environmental and Industrial Biotechnology Group, Pontificia Universidad Javeriana, Bogota 110231, Colombia; acarrasc@javeriana.edu.co; 3 Food Safety Manager V&N Solutions, Bogota 110231, Colombia; apvaron@gmail.com

**Keywords:** microbial profile, *Salmonella*, chicken processing, antimicrobial resistance, Colombia

## Abstract

The poultry industry in Colombia has implemented several changes and measures in chicken processing to improve sanitary operations and control pathogens’ prevalence. However, there is no official in-plant microbial profile reference data currently available throughout the processing value chains. Hence, this research aimed to study the microbial profiles and the antimicrobial resistance of *Salmonella* isolates in three plants. In total, 300 samples were collected in seven processing sites. Prevalence of *Salmonella* spp. and levels of Enterobacteriaceae were assessed. Additionally, whole-genome sequencing was conducted to characterize the isolated strains genotypically. Overall, the prevalence of *Salmonella* spp. in each establishment was 77%, 58% and 80% for plant A, B, and C. The mean levels of Enterobacteriaceae in the chicken rinsates were 5.03, 5.74, and 6.41 log CFU/mL for plant A, B, and C. Significant reductions were identified in the counts of post-chilling rinsate samples; however, increased levels were found in chicken parts. There were six distinct *Salmonella* spp. clusters with the predominant sequence types ST32 and ST28. The serotypes Infantis (54%) and Paratyphi B (25%) were the most commonly identified within the processing plants with a high abundance of antimicrobial resistance genes.

## 1. Introduction

The poultry industry in Colombia has grown steadily in the last ten years, favoring the investment in new processing technologies, genetic selection, good agricultural and manufacturing practices and harmonization with international standards. Additionally, Colombian producers have implemented biosecurity measures to prevent the dissemination of Newcastle and salmonellosis to meet the demands of national and global chains [1]. In 2014, the National Administrative Department of Statistics (DANE, Departamento Administrativo Nacional de Estadística) in collaboration with the National Federation of Poultry Producers of Colombia (FENAVI, Federación Nacional de Avicultores) reported that the valorization of the poultry sector in Colombia was estimated to be 14.8 billion Colombian pesos (4.79 million US Dollars), a record value in the poultry industry.

The Ministry of Agriculture and Rural Development, together with the Ministry of Health and Social Protection, established the Decree 1500 in 2007 to improve food safety programs in the meat and poultry sectors. This decree has been updated in recent years, and in 2016 the national government implemented the last phase of the sanitary laws (Decree 1500 of 2007, Decree 2270 of 2012, and Decree 1282 of 2016, resolution 240/2013), which target the reduction of foodborne illness and the implementation of food safety programs in meat and poultry productions. These updated decrees establish new microbiological verification programs for the conventional inspection system for meat and meat products.

In Colombia, chicken production is concentrated in five-Departments, Santander, Cundinamarca, Valle del Cauca, Cauca, and Antioquia, providing more than two-thirds of the country’s total production [2]. The poultry processing plants in Colombia are classified into two categories: industrial plants and special plants. The first produces more than 3000 birds/day, while the second presents a lower capacity. Industrial plants comprise 97% of the total production volume [3]. There are 118 authorized chicken slaughtering establishments in the country, and 397 more operate under provisional authorization based on the national sanitary standards [4]. In 2016, ten plants were closed because they did not meet the requirements of Decree 1500, while 49% (70/142) followed the general sanitary regulations. 

The National Health Institute (INS, Instituto Nacional de Salud) of Colombia reported 11,502 foodborne illness cases related to 881 outbreaks in 2018 [5]. The Valle del Cauca presented the highest numbers of outbreaks (124/881), followed by Bogota D.C. (78/881) and Sucre (64/881) [5]. *Salmonella* spp. contamination of foods was identified as the attribution source for 29% (159/547) of the total number of outbreaks where the food or surface sample was available. The main food products linked with these outbreaks were cheese (19.4%), chicken meat (10.7%), mixed foods (9.6%), fast food (9.65%), fish and seafood (7.8%), beef and pork meat (6%), and fruit juices (2.6%) [5].

The INS initiated in 1997 a laboratory-based surveillance program to track *Salmonella* spp. clinical isolates around the country. In 2019, the institution reported 20 years of data from different health systems, with 12966 *Salmonella* spp. isolates from the 32 departments. From those isolates, more than 60% were recovered from the Antioquia department (26.4%; 3421/12966), Bogota D.C. (24.5%; 3182/12966), and Valle del Cauca (8.7%; 1127/12966). The top five serotypes identified were Typhimurium (27.6%), Enteritidis (27.1%), Typhi (11.4%), Dublin (3.3%), and Infantis (2.03%). Antimicrobial susceptibility testing of 5185 *Salmonella* spp. isolates was also included in the program from the period 2014–2018, where *S*. Enteritidis (24%; 1247/5195) and *S*. Typhimurium (21%; 1090/5195) presented the highest levels of resistance to ampicillin, cefotaxime, ceftazidime, ciprofloxacin, chloramphenicol, tetracycline and trimethoprim-sulfamethoxazole [6]. However, *Salmonella* spp. infection is an important concern for public health authorities in Colombia. There is no available data on illness source attribution to estimate common causes and determine opportunities to improve the health system. 

Furthermore, there is no public document that collects together information on all the monitoring programs established in the processing plants. The National Institute of Food and Drug Surveillance (INVIMA, Instituto Nacional de Vigilancia de Medicamentos y Alimentos) and FENAVI initiated a national data collection of the prevalence of *Salmonella* spp. and *Campylobacter* spp. in chicken carcasses, and the common serovars found in processing environments. However, this information has not yet been published. Additionally, the INS and the Ministry of Health and Social Protection issued food safety risk profiles for *Salmonella* spp. [7], and *Campylobacter* spp. [8] associated with whole chicken carcasses and chicken parts, due to the increased number of foodborne illnesses in the country. These documents presented a detailed analysis of the risk factors associated with poultry production and processing in Colombia. They also identified the lack of microbial profiling, pathogens serotyping, and antimicrobial resistance surveillance in the design of science-based approaches to control and reduce foodborne pathogens in the poultry industry. 

Hence, this study’s main objectives were to determine the microbial profile during chicken processing operations to assess pathogen prevalence, Enterobacteriaceae levels, and evaluate the genotypic characterization of antimicrobial resistant *Salmonella* spp. strains.

## 2. Materials and Methods 

### 2.1. Characteristics of the Poultry Processing Plants

**Figure 1:**
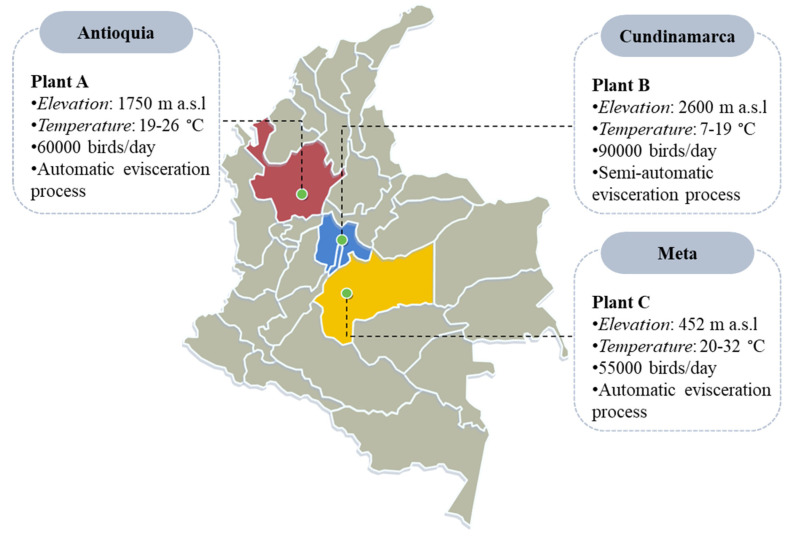
Characteristics of the three poultry processing facilities included in this study.

Three commercial poultry processing plants were sampled between July-October 2017. A summary of the locations and characteristics of each plant is presented in Figure 1. All three establishments utilize 10 ppm hypochlorous acid (HClO) as a chemical intervention in the immersion chiller tank, and tap water for washers and drench applications. They run two schedule shifts of 8 h/day for 5 days per week. 

The broiler chickens processed in the poultry establishments were fed with a diet based on maize and supplement with salinomycin and narasin as a coccidiostat. Subtherapeutic levels of antibiotics such as enramycin, avilamycin, bacitracin, and chlorhydroxy-quinoline were added to the poultry feed as a growth promoter. Therapeutic levels of chlortetracycline, tiamulin, and tilmicosin were also used to treat disease in chickens.

### 2.2. Experimental Design

In total, 270 chicken rinsate and 30 fecal samples were collected in the three plants (100 samples from seven sites in each plant) during a normal processing day. The processing sites were selected based on the production line at the processing plants. Samples were collected at the arrival station (fecal), pre-scalding (after bleeding and before entering the scalding tank), post-scalding (immediately after exiting the scalder tank), post-IOBW (inside-outside body wash), pre-chiller (before entering the pre-chiller tank), post-chiller (immediately after exiting the immersion chill tank), and parts (reconstruct a chicken carcass with legs, breasts, and wings). Plant B did not have a chicken cut-up and deboning room installed. Hence, post-defeathering samples were collected instead of chicken parts. The prevalence of *Salmonella* spp. and the levels of Enterobacteriaceae in chicken rinsate were evaluated for all samples collected.

### 2.3. Chicken Carcass Rinsate Sample Collection

Whole chicken carcass and chicken part rinsates (one full chicken breast, two chicken wings, and two chicken legs) were collected at different processing sites in all three processing plants, according to the USDA-FSIS method MLG 4.09. Briefly, as described in Ramirez-Hernandez et al., [9], chicken carcasses and chicken parts were randomly selected and removed from the processing line and aseptically placed in individual sterile poultry rinse bags (Nasco, Fort Atkinson, WI, USA). Then, 400 mL of sterile buffered peptone water (BPW, BD, Detroit, MI, USA) was added to each bag and distributed by shaking for 1 min. After collection, samples were kept at 5 °C in a cooler and sent overnight to the Pontificia Universidad Javeriana in Bogota, D.C., Colombia.

### 2.4. Fecal Sample Collection

Sampled birds were scheduled to be processed as the first lots on the sampling day. For plant A, fecal samples were pooled by collecting aseptically 25 g from arriving cages (*n* = 10) into a sterile sampling bag (Whirl-Pak; Nasco, Fort Atkinson, WI, USA). For plants B and C, fecal samples were extracted aseptically from the cloaca of individual chickens (*n* = 10) into a sterile sampling bag. Samples were kept 5 °C in a cooler and shipped together with the rinsate samples to the Pontificia Universidad Javeriana in Bogota, D.C., Colombia.

### 2.5. Microbiological Analysis

The microbial analysis was conducted at the food microbiology laboratory in the Department of Microbiology at the Universidad Javeriana in Bogota D.C., Colombia. 

#### 2.5.1. *Salmonella* spp. Detection and Isolation

The prevalence of *Salmonella* spp. was evaluated using a molecular detection system (MDS100, 3M^TM^; St. Paul, MN, USA) with method AOAC 2013, following manufacturer’s instructions. Briefly, for *Salmonella* spp. detection (MDA2SAL96-3M^TM^ molecular detection assay 2-*Salmonella*; St. Paul, MN, USA), 30 mL of rinsate sample were mixed with 30 mL of double strength BPW; fecal samples were diluted and homogenized thoroughly for 2 min and incubated at 37 ± 1 °C for 24 h. The enrichments were transferred to lysis tubes and heated at 100 ± 1 °C for 15 min. Lysates were transferred to a reagent tube, loaded into a molecular detection speed loader tray (3M^TM^), and analyzed using molecular detection software. Presumptive positive samples were then cultured based on the Microbiology Laboratory Guidebook MLG 4.09 (*Salmonella*). Briefly, an aliquot of 0.5 mL of each enriched sample was transferred to 10 mL of Tetrathionate (TT) broth (BD; Sparks, MD, USA), and 0.1 mL were transferred into 10 ml modified Rappaport-Vassiliadis (mRV) broth (BD; Sparks, MD, USA), and incubated at 42 ± 1 °C and 37 ± 1 °C for 24 h. After incubation, tubes were vortexed, and one loopful (10 µL) of the enrichment was streaked on Brilliant Green (BG) agar (Acumedia; Lasing, MI, USA) and Xylose Lysine Tergitol^TM^ 4 (XLT4) agar (EMD; Billerica, MA, USA), and incubated at 37 ± 1 °C for 24 h. *Salmonella* presumptive colonies were selected based on typical morphology and color. Subsequently, the screening slants media Triple Sugar Iron (TSI) agar (BD; Sparks, MD, USA) and Lysine Iron Agar (LIA) (BD; Sparks, MD, USA) were used by stabbing the butts and streaking the slants a single pick colony. Tubes were incubated at 37 ± 1 °C for 24 h, and typical reactions on TSI and LIA slants were evaluated.

#### 2.5.2. Enumeration of Enterobacteriaceae

Dilutions prepared for *Salmonella* detection were used to quantify the levels of Enterobacteriaceae in the chicken carcass and chicken parts rinsates and fecal samples, following the MLG 3.02 protocol (Quantitative Analysis of Bacteria in Foods as Sanitary Indicator). Further, serial diluted (1:10) in BPW were done, and 1 ml of the corresponding dilution was transferred onto Enterobacteriaceae Petrifilm plates (3M^TM^, St. Paul, MN, USA), which were incubated at 35 °C for 24 h, following manufacturer’s instructions.

### 2.6. Whole Genome Sequencing (WGS)

The confirmed *Salmonella* spp. strains were sequenced following the methodology explained by Ramirez-Hernandez et al., [9,10]. WGS analysis was conducted as part of the GenomeTrakr Project: Texas Department of State Health Services. The genomic data of each sequenced strain have been deposited in National Center for Biotechnology Information (https://www.ncbi.nlm.nih.gov/ (accessed on 24 February 2021)) under the Bioproject accession number PRJNA284276. Among the 85 *Salmonella* genome assemblies, there was a mean of 56x (min: 32, max: 103), 4.91 Mbp of genome size (min: 4.51, max: 5.10), and 120.60 of number of contigs (min: 24, max: 279).

### 2.7. Bioinformatic Analysis 

Fastq files containing the sequence data were used to perform contig assemblies using Patric (http://www.patricbrc.org (accessed on 24 February 2021)). Briefly, assembled contig files were run into different pipelines available at the Center for Genomic Epidemiology website (http://www.genomicepidemiology.org/ (accessed on 24 February 2021)), including ResFinder 3.0, SeqSero 1.2, MLST 1.8, PlasmidFinder 1.3, SpeciesFinder 1.2 16S rRNA, and CSI phylogeny 1.4. Lastly, bacterial populations were defined based on the genotypic characteristics of the strains.

### 2.8. Statistical Analysis 

Chi-square test was used to determine the statistical relationship of *Salmonella* spp. prevalence between the processing sites. Enterobacteriaceae counts were analyzed with a non-parametric test Kruskal-Wallis followed by pairwise multiple comparisons post hoc Conover’s tests to identify the significant variation in microbial level on fecal and rinsate samples collected in different sites. A *p*-value of 0.05 was selected for a significant difference in this study.

## 3. Results

### 3.1. Salmonella spp. Prevalence

*Salmonella* spp. were recovered from chicken carcass, chicken parts rinsates and fecal samples at different processing sites (Table 1). The overall prevalence of *Salmonella* spp. in each plant was: Plant A, 77% (77/100; CI, 68 to 84%), Plant B, 58% (58/100; CI, 48 to 67%), and Plant C, 80% (80/100; CI, 71 to 87%).

Plant A: The prevalence of *Salmonella* spp. was unchanged from the post-IOBW processing sites, to 100% (CI, 75–100%) presumptive positive samples (*p* > 0.05). However, samples from the pre-scalding site exhibited a different prevalence than the subsequent processing sites (*p* = 0.001). 

Plant B: Different profiles were presented; *Salmonella* spp. were detected at various levels throughout the process. After pre-chilling, the prevalence was 47% (7/15; CI, 22 to 73%) and a higher level of contamination was detected in chicken parts with 80% (13/15; CI, 58 to 98%) of presumptive positive samples; however, there were no significant differences among the processing sites (*p* = 0.17).

Plant C: 80% (12/15; CI, 51–97) of *Salmonella* spp. positive samples were presented at the post-chilling site, with no statistical differences between processing sites (*p* = 0.29). 

### 3.2. Enterobacteriaceae Levels

**Figure 2:**
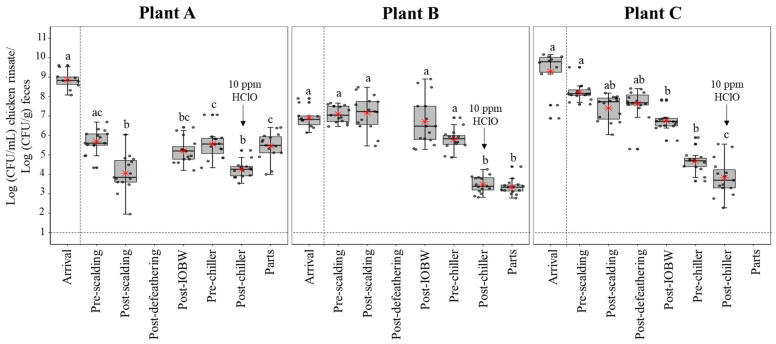
*Enterobacteriaceae* (Log CFU/mL for chicken rinsates; Log CFU/g for fecal samples) levels recovered from the chicken carcass, chicken parts, and fecal samples by each processing plant at the different processing sites. In each box plot, the heavy horizontal line crossing the box is the median, the bottom and top of the box are the lower and upper quartiles, and the whiskers are the maximum and minimum values of the date set. The red star inside the box represents the mean value. Boxes with different letters ^a, b, c, d^ are significantly different according to a non-parametric Kruskal-Wallis followed pairwise multiple comparison post hoc Conover’s test at *p* < 0.05. The grey points represent the actual data points. There are some that appears as darker points is because there are more than one data point with the same or close value.

Enterobacteriaceae levels are presented in Figure 2.

Plant A: Fecal samples at the arrival location presented a mean of 8.85 Log CFU/g (CI, 8.51 to 9.19 Log CFU/g). From the first processing site evaluated (pre-scalding) to the final process (parts), counts of Enterobacteriaceae differed by 0.24 Log CFU/mL, but there were no statistically significant differences (*p* = 0.33). However, there was a considerable difference between Enterobacteriaceae levels in pre-chilling, compared to chicken parts (*p* = 0.003).

Plant B: Fecal samples at the arrival location presented a mean of 6.90 Log CFU/g (CI, 6.51 to 7.29 Log CFU/g). There was a significant reduction for chicken parts (*p* < 0.00001) of 3.76 Log CFU/ml from the pre-scalding. Likewise, the pre-chiller and post-chiller counts presented significant differences with a *p* = 0.017. Nevertheless, there was no statistically significant difference between pre-chiller and chicken parts (*p* = 0.29).

Plant C: Fecal samples at the arrival location presented a mean of 9.30 Log CFU/g (CI, 8.47 to 10.1 Log CFU/g). Similarly to plant B, there was a significant reduction in the Enterobacteriaceae counts from pre-scalding (8.21 Log CFU/mL) to chicken parts (3.83 Log CFU/mL). There was a significant difference between pre-chiller and post-chiller levels with a *p* = 0.01; however, no differences were found when comparing levels in post-chiller and chicken parts (*p* = 0.21).

### 3.3. WGS of Salmonella spp. Isolates

From a total of 215 *Salmonella* spp. positive isolates, only 85 isolates were fully characterized by whole genome sequence analysis. 

Table 2 presents a summary of the *Salmonella* spp. serotypes identified from the processing sites in the three processing plants. In plant A, all 11 isolates were confirmed as *S*. Infantis. In plant B, the serotype Paratyphi B was the most common identified (54%, 14/26), followed by the serotypes Javiana (11.5%, 3/26), Typhimurium (11.5%, 3/26), Heidelberg (3.8%, 1/26), Infantis (3.8%, 1/26), and unrecognized serotypes *?:b:1,2* (3.8%, 1/26), *9:,z28:1,2* (3.8%, 1/26), and *9:-:1,2* (3.8%, 1/26), and an unknown (3.8%, 1/26). From the previous serotypes, they were identified with closely related strains. In the case of *?:b:1,2* which theO antigen was undetermined and the unknow serotype, they were both highly related to an identified *S.* Paratyphi strain with six single different nucleotide polymorphisms (SNPs); similarly, *9:,z28:1,2* with a *S.* Javiana strain differed in 6 SNPs, and the *9:-:1,2*, which missed the phase 1 H antigen, was closely related with a *S*. Infantis strain with three different SNPs. In plant C, 48 isolates were sequenced where the serotype Infantis was confirmed in 73% of the samples (35/48), followed by Paratyphi B (12.5%, 6/48), Javiana (12.5%, 6/48) and *9:-:1,5* (2%, 1/48). The last serotype, missing the phase 1 H antigen was closely related to a *S.* Javiana strain differing in four SNPs. Clonal populations were characterized based on the combined analysis of phylogeny, serotype, multi-locus sequence type (MLST), plasmid incompatibility type, and genotype antimicrobial resistance (AMR) profiles. The sequence types identified among all *Salmonella* serotypes were ST32 (48/85), ST28 (25/85), ST24 (6/85), ST19 (3/85), ST1674 (2/85), and ST15 (1/85).

**Figure 3:**
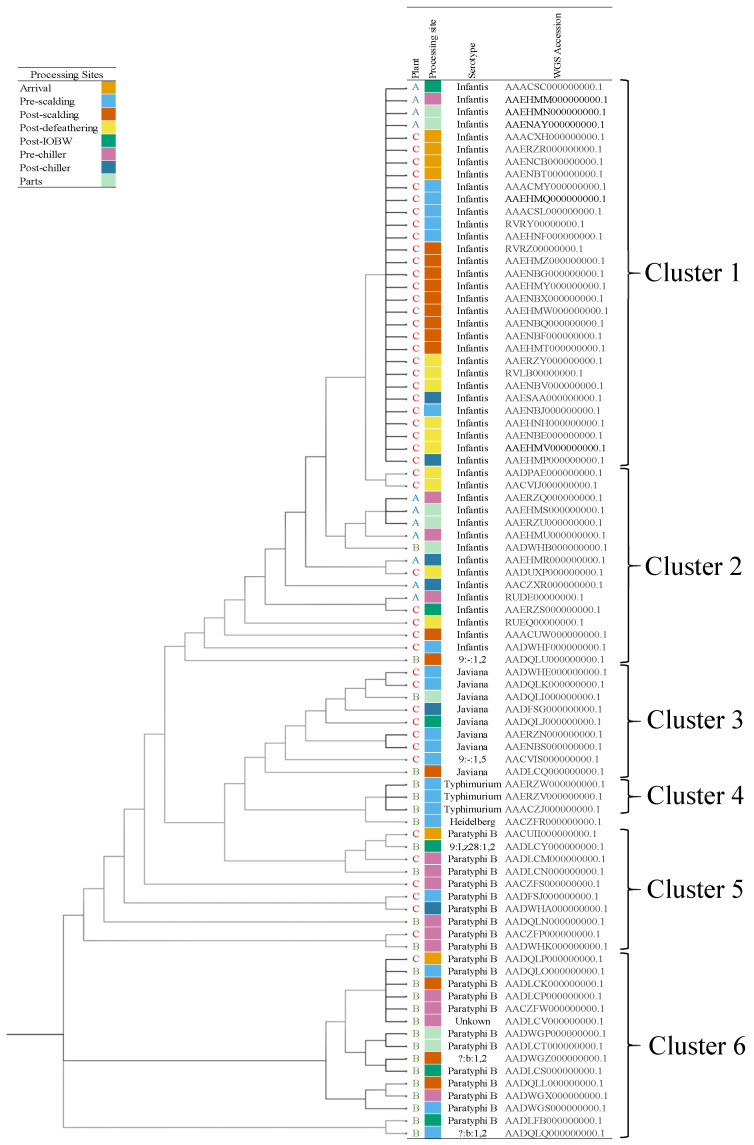
Phylogenetic tree of *Salmonella* spp. strains isolated from the poultry processing plants defined by the concatenated alignment of the high-quality single nucleotide polymorphisms (SNPs). The description in the column represents the plant name, processing site, serotype, and the genome accession number from the NCBI (GenomeTrakr Project: Texas Department of States Health Services, SRA. study: SRP059203, Bioproject: PRJNA284276).

**Figure 4:**
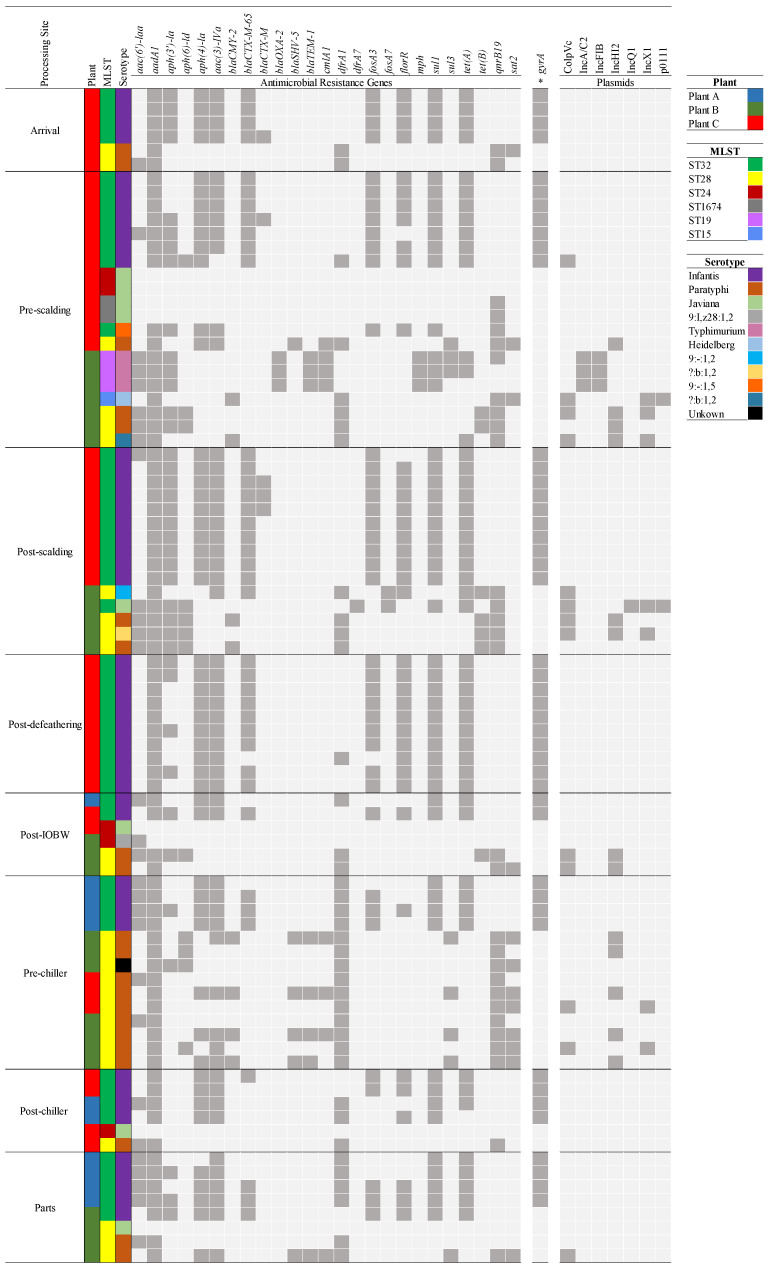
Distribution of antimicrobial resistance (AMR) genes and plasmids of *Salmonella* spp. strains recovered from the poultry processing plants at the differing sites. Colors indicate processing plant, serotype, and MLST. The asterisk represents a chromosomal point mutation within the target genes. Dark grey and light grey show the presence and ab-sence of genes and plasmids in the bacterial genome.

Figure 3 presents the phylogenetic trees of all *Salmonella* spp. strains recovered from the processing plants, created based on the concatenated high-quality single nucleotide polymorphisms, performed using CSI phylogeny 1.4 pipeline (Center of Genomic Epidemiology website). Figure 4 presents the distribution of AMR genes and plasmids of all *Salmonella* spp. strains recovered from the plants at the differing sites. 

Plant A: Two clusters attributed to the serotype Infantis were identified among the *Salmonella* spp. isolates. Although all presented the same sequence type ST32, they differed in the AMR genotypic profile. Strains from the Infantis cluster 1 (4/11) harbor multiple AMR genes *aac(6′)-laa*, *aadA1*, *aph(4)-la*, *aac(3)-IVa*, *dfrA1*, *fosA*, *sul1*, and *tet(A)*. The Infantis cluster 2 (7/11) presented the same eight AMR genes profile as cluster 1, but four (4/6) of the clonal population harbored additional genes, *bla_CTX-M-65_*, *fosA*, and *aac(3)-laa*, and an extra *florR* gene in two (2/4). All strains presented aminoglycosides and aminoglycoside transferase genes (*aac(6′)-laa*, *aadA1*, *aph(4)-la*, *aac(3)-IVa*) which confer resistance to streptomycin and spectinomycin. Moreover, dfrA1 gene conferred resistance to trimethoprim (integron-encoded dihydrofolate reductase), *fosA3* is identified as a Fosfomycin resistance gene, sul1 confers sulfonamide resistance, and *tet(A)* is associated with the resistance to tetracycline. The *bla_CTX-M-65_* gene is correlated with resistance to beta-lactam antibiotics, and *florR* gene is typically associated with intrinsic resistance to phenicol antibiotic class. A chromosomal point mutation was present in all isolates with *gyrA* gene, which is the primary cause of quinolone resistance (nalidixic acid and ciprofloxacin). No plasmids were found on the bacterial genome of the isolates from this processing site.

Plant B: *Salmonella* isolates were classified between clusters 2–6 (Figure 3), except the serotype Heidelberg, of which just one isolate was identified from a pre-scalding rinsate sample. Eleven Paratyphi B strains were categorized in cluster 6, and the most frequent AMR genes among all were *qnrB19*, *aadA1*, and *dfrA1*, which confer resistance to quinolone, aminoglycoside, and trimethoprim. Five strains presented the *bla_CMY-2_* gene associated with the beta-lactam resistance; moreover, two *?:b:1,2* (para-typhic B variant, lacking the O-4 antigen) strains isolated from pre- and post-scalding sites were in cluster 6, and harbor five AMR genes, with an additional *bla_CMY-2_* gene in the strain isolated from the post-scalding tank. Three Paratyphi B strains were assigned to cluster 5, two of the strains were isolated from the pre-chilling tank and carry four AMR genes (*aadA1*, *aph(6)-laa*, *dfrA1*, and *qnrB19*), and one strain isolated from the post-IOBW presented eight genes more (*aac(3)-IV*, *aadA2*, *aph(4)-la*, *bla_CMY-2_*, *bla_SHV-5_*, *cmlA1*, and *sul3*). In addition, one *9:I,z28:1,2* strain isolated from the post-IOBW was also classified in cluster 5 and presented multiple AMR genes (*bla_CMY-2_*, *qnrB19*, *tet(B)*, *aadA1*, *aph(6)-ld*, *aph(3″)-lb*, and *dfrA1*). Three *S*. Typhimurium strains isolated from the pre-scalding site were grouped in cluster 4; they harbor between 17 to19 AMR genes, among them four aminoglycoside resistance genes, *aph(3″)-la*, *aac(3)-lld*, *aadA1*, and *aadA2*, three sulfonamide resistance genes, *sul1*, *sul2*, and *sul3*, two beta-lactam resistance genes *bla_OXA-2_*, and *bla_TEM-1-B_*, two tetracycline-resistant genes, *tet(A)* and *tet(M)*, two trimethoprim resistance genes, *dfrA29* and *dfrA12*, two macrolide resistance gene *mph* and *Inu(F),* a chloramphenicol resistance gene *cmlA1*, and a quinolone resistance gene, *qnrB19*. Next, two *S*. Javiana were in cluster 3, one of them isolated from chicken parts rinsate which did not have AMR gene in its genome; on the other hand, the second Javiana strain isolated from the post-scalding site carried ten AMR genes, including a beta-lactam resistance gene *bla_TEM-1_* and *qnrB19* previously described. Finally, one *S.* Infantis and one *9:-:1,2* (monophasic variant, lacking the expression of flagellar phase 1) strains were identified in cluster 2, both presenting ten AMR genes in common that conferred resistance to aminoglycosides, beta-lactams, trimethoprim, phenicol, sulfonamides, Fosfomycin, and tetracycline; but the strain *9:-:1,2*, harbors the *qnrB19* gene, and extra *aph(3″)-lb*, *aph(6)-lb*, *aac(6′)-laa*, and *tet(B)* genes. 

Plasmid incompatibility types were identified in 20 out of the 26 *Salmonella* spp. strains; the most common plasmids among the *S.* Parathypi B and the variant *?:b:1,2* were IncHI2 (13/20) and ColpVC (10/20); *S.* Heidelberg carry the same plasmids with the addition of IncX1. All *S.* Typhimurium carry the IncFIB and IncA/C2 plasmids. The *S.* Javiana strain isolated from the post-scalding tank has IncQ1, IncX1, ColpVC, and p0111 plasmids.

Plant C: Twenty-seven *S*. Infantis were classified in cluster 1, strains were isolated from different processing sites (arrival, pre- and post-scalding, post-defeathering, and post-chiller), the common resistance genes in all strains were *aadA1*, *aph(4)-la*, *aac(3)-IV*, *sul1*, and *tet(A).* Furthermore, 26 out of 27 strains harbored the *fosA3* gene, and 25 of them carry the *bla_CTX-M-65_* and *florR* genes. Besides, five *S*. Infantis were categorized in cluster 2; they were characterized to have the *aadA1*, *aph(4)-la*, *aac(3)-IVa*, *bla_CTX-M-65_*, *fosA3*, *florR1*, *sul1*, and *tet(A)* genes, and four had an extra *aph(3’)-la* gene. A chromosomal point mutation (*gyrA* gene) was presented in all *Salmonella* Infantis strains. Furthermore, seven *S*. Javiana strains were classified in cluster 3, and five of these were isolated from the pre-scalding, one from post-IOBW, and one from the post-chiller tank. Only two carry AMR genes associated with quinolone resistance (*qnrB19*). Five *S*. Paratyphi B strains were categorized in cluster 5, all carrying the *dfrA1*, *aadA1*, and *qnrB19* genes, and two strains isolated from the pre-scalding site harbor seven genes more, *aac(3)-IV*, *aadA2*, *aph(4)-la*, *bla_SHV-5_*, *bla_TEM-1_*, *cmlA1*, and *sul3*. One *S*. Paratyphi B strain isolated from the arrival site was grouped in cluster 6, and had *aac(6′)-laa*, *aadA1*, *qnrB19*, and *dfrA1* genes.

ColpVC plasmid was found in one out of the 34 *S*. Infantis strains isolated from the post-scalding tank. *S*. Paratyphi B strains (2/8) carry the plasmids ColpVC and IncX1 and one (1/8) also carries IncH12 and Incl1 plasmids.

### 3.4. The Relative Abundance of AMR Genes within Processing Sites 

**Figure 5:**
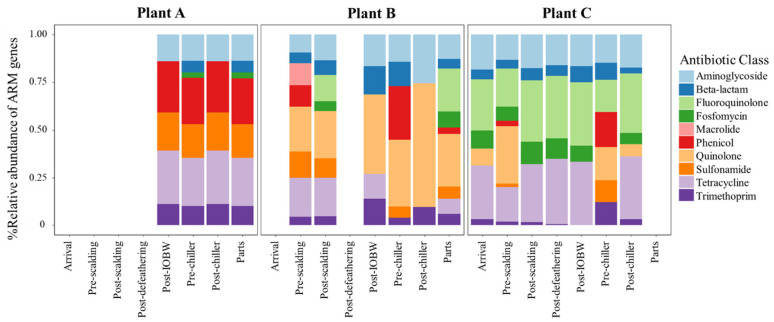
The relative abundance percentage of antimicrobial resistance genes classed into antibiotic classes among the processing sites.

Figure 5 shows the relative abundance percentage of the most-abundant AMR genes grouped by the antibiotic class they provide resistance to.

Plant A: AMR genes were grouped in the following antimicrobial classes: aminoglycoside, beta-lactam, Fosfomycin, phenicol, sulfonamide, tetracycline, and trimethoprim. There were no statistically significant differences (*p* > 0.05) in the abundance of AMR genes classified in the antimicrobial families within the processing sites (post-IOBW, pre-chiller, post-chiller, and parts) where the strains were isolated. 

Plant B: The abundance of AMR genes changed throughout the stages of processing. There was a statistically significant difference in the abundance of aminoglycoside resistance genes in the processing sites post-scalding (*p* = 0.0247), pre-chiller (*p* = 0.0217), and pre-scalding (*p* = 0.0126) compared with chicken parts. Moreover, there were differences in the relative abundance of beta-lactam AMR genes from the pre-chiller and parts (*p* = 0.0134), pre-chiller, and post-chiller (*p* = 0.0076) sites. The abundance of macrolide resistance genes was characterized only in five strains isolated from the pre-scalding site; hence, there were statistical differences with the following sites. Similarly, variation in the abundance of sulfonamide resistant genes was identified from pre-scalding compared with pre-chiller (*p* = 0.0073), post-chiller (*p* = 0.0036), and chicken parts (*p* = 0.002). The abundance of tetracycline resistance genes presented significant differences from the pre-scalding site contrasted with the abundance in chicken parts (*p* = 0.0015), pre-chiller (*p* = 0.0007), and post-chiller (*p* = 0.0063). There was no statistically significant difference in the abundance of trimethoprim resistance genes within the processing sites.

Plant C: The isolates recovered from this processing plant presented the highest variability and abundance of AMR genes. There were statistically significant differences in the abundance of aminoglycoside resistance genes in the post-scalding site compare with the arrival (*p* = 0.0011), post-chiller (*p* = 0.0001), and post-IOBW (*p* = 0.0063); the pre-chiller site also had the lower abundance of resistance genes. There were considerable differences within post-defeathering (*p* = 0.009), post-scalding (*p* = 0.0005), and pre-scalding (*p* = 0.0007). 

Genes associated with Fosfomycin resistance presented significant differences in the pre-chiller and post-chiller sites compared with post-defeathering (*p* = 0.0232 and 0.0232) and post-scalding (*p* = 0.0098 and 0.0098). There was also a difference between the abundance during pre-scalding and post-scalding (*p* = 0.0168). The abundance of fluoroquinolone was significantly lower in arrival (*p* = 0.0061), pre-chiller (*p* = 0.0052), post-chiller (*p* = 0.0026), and post-IOBW (*p* = 0.00148) compared with post-scalding site; besides, there was statistical significance in post-defeathering and post-chiller (*p* = 0.0224). Phenicol resistance genes were present only in the pre-scalding and pre-chiller sites, and there was a significant difference (*p* = 0.0023). There were considerable differences between the abundance of tetracycline resistance genes from post-scalding site compared with arrival (*p* = 0.0057), pre-scalding, (*p* = 0.0007), post-IOBW, (*p* = 0.0034), pre-chiller (*p* = 0.0040), and post-chiller (*p* = 0.0304). In addition, the relative abundance in post-defeathering differed from pre-scalding (*p* = 0.0102), pre-chiller (*p* = 0.0024), and post-chiller (*p* = 0.0204). Trimethoprim resistance genes were present in all processing sites except in pre-chiller, and there were no statistically significant differences among the other sites. Genes associated with quinolone resistance were statistically different in pre-chiller contrasted with post-scalding (*p* = 0.0043) and post-defeathering (*p* = 0.0043). There was no statistically significant difference in the abundance of beta-lactam and sulfonamide resistance genes within the processing sites.

## 4. Discussion

In this study, the prevalence of the primary poultry-associated pathogen was assessed during processing. *Salmonella* spp. are commonly detected at high rates throughout processing, including after the chilling operation with a prevalence of 100%, 47%, and 87%, respectively, for plants A, B, and C. It could be inferred that the application of 10 ppm of HClO is not enough to reduce the microbial load in the chicken carcasses. Besides, for chloride to be efficient, the pH of the water must remain in a range of 5.8 to 6.8 [11], and increasing of water pH and organic load will decrease its efficacy [12,13]. The poultry processing plants sampled were characterized by measuring the pH and chloride concentration in the first shift of the processing day, moreover, having a counterflow immersion chiller that moves toward the chicken carcasses to the cleanest water. However, during the day, organic load accumulation is likely to occur in the chiller tank, causing either direct contamination or cross-contamination with *Salmonella* spp. [14].

On the other hand, the levels of Enterobacteriaceae were quantified throughout the processing. Overall, there were significant reductions in the counts of Enterobacteriaceae from the pre-scalding to the post-chilling sites. Nevertheless, in plant A, samples from chicken parts exhibited higher Enterobacteriaceae levels than in previous stages (post-chiller). In the process of cutting the chicken carcass into chicken parts, which usually requires the intervention of experienced workers, handling and manipulation increased the chances of cross-contamination. Bacterial contamination can occur from equipment surfaces, water, and animal microbiota; microorganisms from the environment and air can contaminate chicken parts [15,16].

In Colombia, few studies have investigated the prevalence of foodborne pathogens throughout processing. Most researchers have focused on retail market scenarios [17,18,19]. Nonetheless, Ramirez-Hernandez et al., [20] reported the prevalence of *Salmonella* in three poultry processing establishments, with a maximum overall rate of 21.2% (51/240 samples) in an establishment located in the Department of Valle; additionally, the numbers of presumptive *Salmonella*-positive samples after chilling were lower (<18%) compared to the results obtained in this study. Differences in food safety programs and manufacturing practices implemented in the processing plants, and other variables such as geolocation and seasonality, could explain the different levels of indicator microorganism and *Salmonella* rates in the chicken carcasses.

Decree 1500 established *Salmonella* spp. as a microorganism when verifying performance standards in processing facilities. Generic *E. coli* is also considered an indicator microorganism to check the control process and cleaning and sanitation procedures. These measures are conducted only in the post chilling operation, and there is no monitoring before and after this site. The cut-up and deboning processing room have been shown to include the most critical steps for cross-contamination of chicken meat. Because chicken parts, comminuted chicken, and seasoned chicken parts have become an emerging product category in Colombia, it is crucial to assess the levels and prevalence of indicator microorganisms and pathogens to design appropriate strategies to ensure product safety before packing. 

AMR has become a global public health concern for both human and animal health [21]. Improper use of antibiotics in animal production and human medicine has been shown to be the leading cause of the emergence of AMR bacteria [22,23,24]. Antimicrobial resistance of poultry-associated pathogens in Colombia is a new field of study that has gained more attention from associated government institutions and research groups in universities. Donado-Godoy et al., [25] are among the pioneers in the Colombian Integrated Surveillance Program for Antimicrobial Resistance (COIPARS) pilot project established in 2013. They reported that 23% (139/600) of the carcass rinsates collected at the post-chilling sites in different processing plants in Colombia were positive for *Salmonella*. Among these isolates, high levels of resistance were presented to ampicillin (64%, 84/132), cefotaxime (57%, 75/132), ceftiofur (58%, 72/125) and ciprofloxacin (85%, 111/131). In the same way, *Campylobacter* spp. was detected in 36% (215/600) of the samples. Isolates exhibited resistance to ciprofloxacin (92%, 70/76) and tetracycline (93%, 71/76). 

Results obtained in this work on the genotypic characterization of *Salmonella* spp. indicate high rates of AMR profiles among isolates. It is considered that the presence of AMR genes can represent the phenotypical resistance of antimicrobial agents. However, there are several mechanisms of antimicrobial resistance in bacteria, and there is not always an association with a specific gene. Moreover, such resistance mechanisms can naturally occur or acquire transferable genetic elements (i.e., plasmids or resistance gene encoding integrons). Cross-resistance to antimicrobials can also occur with resistance to different group members of chemical-related components, and/or with the same or similar mechanism of action [26,27]. The presence of *qnrB19* gene in the *Salmonella* genomes (36%, 31/85) of the isolates collected in this study is associated with quinolone resistance and could be attributed to the extensive use of chlohydroxy-quinoline (quinolone class) in the feed to promote the healthy growth of the chickens.

More extensively, research work has been conducted on antimicrobial resistant bacteria from retailed chicken meat in Colombia. Donado-Godoy et al., [18] reported high levels of resistance to tilmicosin (100%, 51/51) and nalidixic acid (66%, 34/51) among *Salmonella* spp. isolates recovered from retailed chicken carcasses in Bogota. Another study conducted by the same group [28] reported the AMR profiles of 378 *Salmonella* spp. isolates from chicken carcasses available in wet markets, supermarkets, and independent markets, collected in six different Departments. Overall, 94% (354/378) of the isolates were resistant to at least one antimicrobial. High levels of resistance were identified for nalidixic acid (70%) tetracycline (57%), streptomycin (67%), and trimethoprim-sulfamethoxazole (54%). Furthermore, 59% presented a multi-drug resistant (MDR) phenotype with resistance to 6 to 15 antimicrobial agents. Additionally, a recent study identified *Salmonella* Heidelberg (3%, 15/540) strains recovered from a poultry processing plant in Santander. Phenotypical characterization indicated *Salmonella* MDR profiles, including resistance to the antibiotic classes of quinolones, fluoroquinolones, cephalosporins, beta-lactams, aminoglycosides, and tetracyclines [29].

The serotype Infantis was the most commonly isolated among the poultry processing plants. *S*. Infantis has emerged as one of the most common serovars causing human salmonellosis in Europe [30] and the United States [31]. An outbreak of MDR *S.* Infantis strain in the United States linked to raw chicken products infected 129 people in 32 states; 21 were hospitalized, and one death has been reported [32]. The outbreak strain was identified in samples from raw chicken products from 76 slaughter and processing establishments, and from live chickens [32]. The increasing prevalence of *S.* Infantis has been characterized by MDR profiles and the harboring of MDR genes, such as the extended-spectrum β-lactamases (ESBLs) [33]. Of the ESBL enzymes, the CTX-family is the most widely reported. Before 2014, the *bla_CTX-M-65_* gene had only been described in *E. coli* isolates from a patient in the United States. In Colombia, the INS reported results of extended-spectrum cephalosporin-resistance markers from *Salmonella* spp. clinical isolates recovered from 1997–2018. The most frequent ESBL genotype identified was the CTX-M (79%; 164/208), especially from the serotypes Typhimurium (40%; 65/164) and Infantis (29%; 48/164) (18). The closely related MDR of Infantis was disseminated among the broiler population and associated with animal production environments, eventually spreading into the food chain and potentially into humans [30,34].

The WGS analysis using ResFinder confirmed the presence of the *bla_CTX-M-65_* in 31 (31/40; 77%) *S.* Infantis isolated from the poultry processing plants in Colombia. All the *bla_CTX-M-65_-* positive isolates carried other resistance genes to aminoglycoside, Fosfomycin, phenicol, sulfonamide, tetracyclines, and trimethoprim. Our results of the genotypic characterization of *S*. Infantis correlate with the profiles of strains isolated from chicken broilers and human cases in Switzerland [30], Great Britain [35], Italy [36], United States [33], and Ecuador [37]. Additionally, Castellanos et al. [38] reported *bla_CMY-2_* and *bla_CTX-M-65_* genes which encode resistance associated with AmpC β-lactamases and ESBLs in both *S*. Paratyphi B (ST28) and *S*. Heidelberg (ST15) from poultry-associated isolates in Colombia. The *bla_CMY-2_* gene was present in nine MDR *S*. Paratyphi B, and *S*. Heidelberg isolates from plant B and C. Similarly, Castro-Vargas et al., [29] showed the presence of *bla_CMY-2_* gene and *bla_CTX-M_* in *S*. Heidelberg isolates recovered from broilers’ cloacal swabs in poultry farms in Colombia.

Furthermore, *S*. Paratyphi B was the second most frequently isolated serotype among all *Salmonella* isolates, and were distributed throughout the different processing sites. Previously published work reported that *S*. Paratyphi B dT+ and *S*. Heidelberg were the most prevalent serotypes isolated from farms and retail meat samples in Colombia during 2008 and 2009 [28]. There were also three *S*. Typhimurium ST19 strains isolated from plant B with a high abundance of AMR genes. This strain ST19 has been recently reported in a study from *Salmonella* spp. clinical isolates (87%; 182/209) in Colombia from 1999 to 2017. These strains were resistant to nalidixic acid and ciprofloxacin, and they are associated with the presence of *qnrB19* genes [39], also identified in our strains. The high rate of AMR genes in the *Salmonella* spp. isolates was associated with multiclass resistance antibiotics. The AMR gene abundance of ten antimicrobial classes were distinct when comparing processing plants, and within processing sites. Understanding the AMR gene profiles of foodborne pathogens could help to predict the risk associated with poultry products’ consumption, target intervention, and limit the dissemination of AMR genes through the food chain. 

The national poultry sector in Colombia faces a variety of challenges motivated by the emergence of fast and dynamic markets that demand better quality, safety, and diversified products. The abundance of AMR *Salmonella* spp. strains circulating in chicken meat represents a public health concern for potential foodborne outbreaks in the country. Hence, data collected at different stages throughout the chicken processing value-chain can help support the implementation of science-based risk management options focused on proven mitigation stages for pathogen control, while ensuring microbial safety in chicken meat products.

## 5. Conclusions

The results of our study identified that Enterobacteriaceae levels decreased throughout the processing sites; however, counts ranging from 3 to 5 Log/CFU/mL rinsate were found after chilling. There was a high prevalence of *Salmonella* spp. in the processing sites and a high rate of AMR genotype among *Salmonella* spp. isolates recovered from the three plants. Serotypes Infantis and Paratyphi B were the most common isolates from the chicken rinsates, and they exhibited different genotypic characteristics. The diversity of AMR genes on the *Salmonella* genomes from the processing sites suggests that changes in their abundance could be attributed to the site conditions, resulting in variations in the natural resistance genes or the acquisition of new AMR genes by mutation or plasmids transfer. Nevertheless, further studies are necessary for identifying emerging or reemerging *Salmonella* serotypes and the routes of contamination in chickens to improve prevention and control methods in poultry processing operations.

## Figures and Tables

**Table 1:** *Salmonella* spp. prevalence from chicken samples collected at different processing sites.

Processing Site	Plant A		Plant B		Plant C	
	No. Positive (%)	95% CI	No. Positive (%)	95% CI	No. Positive (%)	95% CI
Fecal material						
Arrival	1/10 (10)	0.5–46	0/1 (0)	0–34	4/10 (40)	14–73
Chicken carcass rinse						
Pre-scalding	2/15 (13) ^a^	23–42	12/15 (80) ^a^	51–97	12/15 (80) ^a^	51–97
Post-scalding	14/15 (93) ^b^	66–99	8/15 (53) ^a^	27–78	15/15 (100) ^a^	75–100
Post-defeathering	-		-		13/15 (87) ^a^	58–98
Post-IOBW (inside-outside body wash)	15/15 (100) ^b^	75–100	6/15 (40) ^a^	17–67	11/15 (73) ^a^	45–91
Pre-chiller	15/15 (100) ^b^	75–100	12/15 (80) ^a^	51–97	13/15 (87) ^a^	58–98
Post-chiller	15/15 (100) ^b^	75–100	7/15 (47) ^a^	22–73	12/15 (80) ^a^	51–97
Parts	15/15 (100) ^b^	75–100	13/15 (87) ^a^	58–98	-	

^a,b^ Prevalence followed by different superscripts is statistically different (Comparison between processing sites). 95% CI: 95% Confidence Interval.

**Table 2:** *Salmonella* spp. serotypes isolated from different processing sites (No. of isolates). SeqSero-1.2 pipeline from the Center of Genomic Epidemiology.

Processing Site	Plant A	Plant B	Plant C
	(*n* = 11)	(*n* = 26)	(*n* = 48)
Arrival			Infantis (4)
			Paratyphi B (2)
Pre-scalding		Typhimurium (3)	Infantis (7)
		Heidelberg (1)	Javiana (4)
		Paratyphi B (2)	*9:-:1,5* * (1)
		*?:b:1,2* * (1)	Paratyphi B (1)
Post-scalding		*9:-:1,2* * (1)	Infantis (10)
		Javiana (1)	
		*?:b:1,2* * (1)	
		Paratyphi B (2)	
Post-defeathering			Infantis (10)
Post-IOBW	Infantis (1)	*9:I,z28:1,2* * (1)	Infantis (1)
		Paratyphi B (2)	Javiana (1)
Pre-chiller	Infantis (4)	Paratyphi B (6)	Paratyphi B (3)
		*Unknown* * (1)	
Post-chiller	Infantis (2)		Infantis (2)
			Javiana (1)
			Paratyphi B (1)
Parts	Infantis (4)	Infantis (1)	
		Javiana (1)	
		Paratyphi B (2)	

* The predicted antigenic profile does not exist in the White-Kauffmann-Le Minor scheme.

## Data Availability

The data presented in this study are available on request from the corresponding author. The data are not publicly available due to their containing information that could compromise the image of the poultry processing plants.
